# Case of Imperforate Urethra

**Published:** 1810-02

**Authors:** 


### *To the Editors of the Medical and Physical Journal.*

Gentlemen,

Medical Men have, in all ages, been eager to place upon record instances of uncommon formation of parts; and very frequently, have attached more importance to such cases, than in reality belongs to them. Many malformations of the human body are merely curious; and by far the greater proportion of the remainder, afford but very scanty additions to our practical information; and those only through the remote paths of physiology and general reasoning. There still, however, remain a few, the knowledge of which will, sometimes, materially assist us in relieving symptoms, whose causes it would, otherwise, be impossible to understand.

It is true, that, in the case of a new-born infant being incapable of making water, a sensible practitioner would examine the external urinary organs; but, perhaps, if he had not heard of the urethra sometimes being found imperforate, he might not think of such an examination, until other means had failed, which would necessarily involve a certain degree of protracted suffering to the child; on this account, I have thought it would be useful to send you the particulars of such an instance, which happened to me a week ago.

A male child, born on the morning of the 3d of this month, was observed to be very cross on the evening of the 4th, and to suffer from great, though unavailing efforts to make water. The nurse supposed it had heretofore made water at the time it went to stool, from its motions being large and mixed with fluid; so that no idea was entertained of the child having an imperforate urethra. The forcing increased in the night, and on the morning of the he 5th, the attention of the bye-standers was drawn towards the external organs, in consequence of the penis appearing much distended; it being, according to the nurse's calculation, as large as half of her little finger, and nearly as hard.

Upon examination, they found a small vesicle projecting at the part where the urethra should have terminated, which soon after, from the child's exertions, burst and discharged a stream of urine with great force; the child was instantly relieved, and passed nearly a small tea-cupful of the natural secretion.

The nurse is positive it was a membrane drawn over the orifice of the urethra, because the distention of the penis had brought the end of the glands nearly on a level with the end of the fore skin, so that she had very little difficulty in completely uncovering the glans.

